# How Menstruation Is Perceived by Adolescent School Girls in Gedeo Zone of Ethiopia?

**DOI:** 10.1155/2020/3674243

**Published:** 2020-08-19

**Authors:** Zelalem Belayneh, Moges Mareg, Birhanie Mekuriaw

**Affiliations:** ^1^Department of Psychiatry, College of Health and Medical Science, Dilla University, Dilla, Ethiopia; ^2^Department of Reproductive Health, School of Public Health, College of Health and Medical Science, Dilla University, Dilla, Ethiopia

## Abstract

**Introduction:**

Perception regarding menstruation is insufficiently acknowledged. Lack of adequate perception towards menstruation may make girls vulnerable to mental, emotional, and physical problems. This might also be a reason for the failure of menstrual hygiene practice which in turn can have multiple social and health consequences.

**Objective:**

To assess the perception and correlation regarding menstruation among adolescent high school girls in Gedeo zone, Southern Ethiopia.

**Methods:**

An institutional-based cross-sectional study was conducted among a randomly selected 791 adolescent high school girls at the Gedeo zone through the multistage sampling technique. Data were collected using an interviewer-administered questionnaire. The data were entered to EPi Info version 3.5 and exported to SPSS version 20.0 for analysis. Frequency tables were used to describe study variables. Odds ratio with 95% confidence interval was computed to determine the level of significance.

**Result:**

From a total of 806 adolescent girls that were invited to participate in the study, 791 (98.1%) participated. The mean (±SD) age of respondents was 16.3 (±4.7) years. Living alone in dormitories {OR = 1.75 CI = (1.07, 2.85)}, lower maternal educational status {OR = 4.03, CI = (2.41, 6.74)}, and age of menarche before 12 years {OR = 2.07, CI = (1.02, 4.24)} were factors statistically associated with unfavorable perception regarding menstruation.

**Conclusion:**

Most high school girls had an unfavorable perception regarding menstruation. Living alone, lower maternal educational status, and age of menarche before 12 years were factors with statistically significant association with unfavorable perception regarding menstruation. This demonstrates a need to design and implement advocacy programs.

## 1. Introduction

Menstruation is typically a universal phenomenon unique to the females during a woman's reproductive age [1]. The onset of menstruation is one of the most important changes occurring among the girls during their adolescent years, which most commonly occurs between 11 and 15 years with a mean age of 13 years [2]. This is the ideal time in which girls join high schools and colleges [3].

The onset of the first menstrual period is a qualitative event of major significance changes in a woman's life, denoting the achievement of major functional states in social-, occupational-, and health-related aspects [4]. The bodily changes associated with puberty will have an impact in the girl's physical, psychological, and social development and self-image. However, most women are uncomfortable in discussing the “topic” as it has a social taboo [5]. Women shared several misconceptions and traditional beliefs regarding menstruation [6]. Young girls experience more misperception and traditional beliefs than adult women [7] as they have no adequate information from the public, families, and peers regarding menses due to social taboo of discussion regarding these issues. The less information they might receive even commonly have some myth contents [8–10].

Lack of adequate perception towards menstruation may make girls vulnerable to mental, emotional, and physical problems, especially during their menstruating days [11–13]. Premenstrual dysphoric syndrome (PMS) symptoms such as depressed mood, sad, lonely, anxious, nervous, mood swings, trouble with relationships, irritable, angry, impatient, difficulty concentrating, feel out of control, cannot cope, less productive in their job or home, and avoiding social activity [14, 15] can also be more common among girls with poor perception of their menstruation. These symptoms impair their daily activities; reduce academic performance, increase school absenteeism, and negatively impact family relation [16, 17].

The manner in which a girl perceives about menstruation and its associated changes may have an impact on her response to the event of menarche [18]. Women having better knowledge and perception regarding menstruation are more likely to have safe menstrual hygiene practice. Hygiene-related practices of women during menstruation are of a considerable importance in women's health and fetus [19]. The poor menstrual hygiene practice increases vulnerability to reproductive tract infections (RTI) and GUT infection [20]. The infection can be transmitted to the offspring of the pregnant mother too [5]. This calls a comprehensive effort to minimize community misperceptions about menstruation right from their adolescent ages [21, 22]. This may help in mitigating the suffering of millions of women [23].

Despite this missed opportunity and its public health significance, perception regarding menstruation has not received adequate attention in many developing countries, especially Ethiopia [24, 25]. Therefore, the current study was aimed to assess the level of perceptions and associated factors regarding menstruation among adolescent high school girls, in Southern Ethiopia.

## 2. Methods

### 2.1. Study Design, Period, and Setting

An institutional-based cross-sectional study was conducted at Gedeo zone high schools from May 1, 2018, to February 1, 2019. In the zone, there are about 25 high schools serving for 2,351 female students.

### 2.2. Sample Size Determination and Sampling Techniques

The assumptions made for sample size calculation were a 95% confidence interval, poor perception of menstruation [25] 39.9% (*p*=39.9%), and a 5% margin of the error. By adding a 10% nonresponse rate and design effects of 2, the total sample size was 806.

First, six high schools were randomly selected from the total of 25 high schools. The calculated sample size was proportionally allocated to the six randomly selected high schools. Then, the systematic random sampling technique was used to select individual participants in each high school through intervals (*K*). The interval (*K*) was calculated for each high school by dividing the total amount of eligible students (age of 13 and above, regular menstruation in the last two consecutive cycles, currently not pregnant, or breast feeding) to the proportionally allocated samples to be drawn from that high school. The first participant was selected by the lottery method between 1 and *K*. Finally, the “*K*” value was added until the proposed samples size was addressed.

### 2.3. Data Collection Instruments and Procedures

An interviewer-administered questionnaire was used for the data collection. The questionnaire had different domains, including sociodemographic profile, perception-related questions, and Oslo 3-item social support scale.

Level of perception was assessed using a series of 20 Likert scale questions adopted from different literatures [8, 11, 26–29]. The questions had a range of scores (0 = strongly disagree, 1 = disagree, 2 = agree, and 3 = strongly disagree) for correct statements and scored reversely for false sentences. Individuals who score equal to or above the mean (50%) of the total scores (60) were considered as having good perception.

The questionnaire was prepared in English and translated to Amharic and Gedeufa (the commonly spoken languages in the study area) and back to English to check its consistency. Pretest was done at the Chuko high school among 5% (40) of female high school students.

Before the data collection, ethical clearance was obtained from the Institutional Review Board (IRB) of Dilla University. Permission letter was also obtained from the Gedeo Zone Educational Bureau. All participants above the age of 16 and the parents/guardians of participants below the age of 16 were requested to provide written consent after a brief explanation about the purpose and objectives of the study. Participants were also informed of their right to refuse or withdraw their participation at any time they want, and they would face no harm due to their refusal. The data were collected by twelve trained psychiatric nurses supervised by four MSc level mental health professionals. Full freedom was given to study participants to refuse or discontinue participation at any time they want. Personal identifiers like name or phone number have never been recorded at the time of data collection, and the collected data were kept confidential.

### 2.4. Data Processing and Analysis

First, the collected data were checked for their completeness and consistency. The data were then entered to Epi Info version 3.5 and exported the Statistical Package for Social Science (SPSS, version 20) for analysis. Descriptive statics were computed to measure the level of perception and distribution of the study variables. Bivariable and multivariable logistic regression was computed to identify factors associated with poor perception regarding menstruation. All variables with a *p* value less than 0.25 in the bivariable logistic regression were entered together into a multivariable logistic regression in order to control the potential confounders. In the multivariable logistic regression analysis, variables with a *p* value of less than 0.05 were considered as statistically significant.

## 3. Result

From a total of 806 adolescent girls invited to participate in the study, 791 (98.1%) participated. The mean (±SD) age of respondents was 16.3 (±4.7) years. Majority (67%) were within the age ranges of 15–18 years. More than half (58.3%) were Gedeo in ethnicity, and 32.2% were living with their parents (Table 1).

### 3.1. Level of Menstrual Perception

There were numerous traditional beliefs and perceptions regarding menstruation among adolescent high school girls in the Gedeo zone. “Disease as a cause of menses” and “Menses as a lifelong process” were most commonly endorsed misconceptions shared by 51.8% and 50.2%, respectively. Generally, about 540 (68.3%) of participants had an unfavorable perception (scored <50% of the total score of the perception related questions) (Table 2).

### 3.2. Factors Associated with Perception towards Menstruation

Both bivariable and multivariable logistic regression analyses were done to assess factors associated with perception towards menstruation. Living alone in dormitories, lower maternal educational status, and age of menarche before 12 years were factors with statistically significant association with unfavorable perception regarding menstruation (Table 3).

## 4. Discussion

There are many traditional beliefs and perceptions shared by adolescent girls regarding menstruation both in developing and developed nations, and the problem becomes higher among adolescent high school girls [8]. The findings of this research work confirm this conclusion. Accordingly, the finding of this study showed that 540 (68.3%) of adolescents high school girls in the Gedeo zone had an unfavorable perception regarding menstruation. This is in line with a study conducted in Southern Ethiopia in which 60.1% of high school girls had poor knowledge regarding menses [25] and in Northern Ethiopia [6] and Nigeria, which states that perceptions regarding menstruation are poor, and practices are often incorrect among young girls [9].

The findings of the current study showed that Gedeo zone high school girls had more unfavorable perceptions than reports of a study conducted in India in which 61.3% of adolescent girls have good awareness about menstruation [28]. However, the findings of this study showed a better perception of high school girls towards menstruation as compared to a similar study conducted in India, which revealed that only 28.7% had knowledge regarding menstruation [8].

This study also assessed the correlation of perception towards menstruation among high school girls. Accordingly, the odds of having poor perception regarding menstruation among girls who are living alone were 1.75 times higher {OR = 1.75 CI = (1.07, 2.85)} as compared to girls who are living with their parents. This finding is parallel to the conclusions of other studies. This might be due to the lack of access to adequate information from their mother, sisters, and other family members [19].

Maternal educational level has a statistically significant association with poor perception of menstruation among high school girls [25]. Accordingly, the current study showed that girls whose mother is unable to read and write were 4.03 times more likely to have poor perception towards menstruation {OR = 4.03, CI = (2.41, 6.74)} as compared to girls whose mother's educational level is diploma and above. This might be explained by the fact that mothers with higher educational levels can discuss with their daughters freely about the truths of menstruation, and mothers with lower educational status might not be open to discuss regarding menstruation or might deliver misperceived ideas [29].

Similarly, girls whose menarche is before 12 years of age were 2.07 times more likely to have poor perception regarding menstruation {OR = 2.07, CI = (1.02, 4.24)} as compared to girls whose menarche was after 15 years. This might be explained as girls whose menarche was starting late can have better opportunity to share good knowledge and prepare and adjust themselves for menstruation than girls whose menarche was stated earlier [3, 21, 29].

## 5. Conclusion

There were numerous traditional beliefs and perceptions regarding menstruation among adolescent high school girls in the Gedeo zone. Most (68.3%) of the high school girls had unfavorable perceptions regarding menstruation. Living alone in dormitories, lower maternal educational status, and age of menarche before 12 years were factors with statistically significant association with unfavorable perception regarding menstruation. This demonstrates a need to design and implement awareness creation and advocacy programs. Special attention is expected to be given to girls who live alone and who have lower maternal educational status and lower age of menarche.

## Figures and Tables

**Table 1:** Sociodemographic characteristics of adolescent high school girls in Gedeo zone, Southern Ethiopia (*N* = 791).

Variable	Categories	Frequency	Percentage
Age in years	<15 years	185	23.4
	15–18 years	530	67.0
	>18 years	76	9.6

Living with	Parents	290	31.3
	Relatives	253	27.3
	Peers	215	23.2

Residency	Rural	548	69.3
	Town	243	30.7

Religion	Protestant	461	58.3
	Orthodox	206	26.0
	Muslim	64	8.1
	Others	60	7.6

Ethnicity	Gedeo	409	51.7
	Oromo	182	23.0
	Amhara	133	16.8
	Guragie	25	3.2
	^*∗*^Others	42	5.3

Birth order	First	285	36.0
	In between	326	41.2
	Last	180	22.8

Maternal education	Unable to read and write	278	35.1
	Able to read and write	138	17.4
	Primary	213	26.9
	Secondary	73	9.2
	Diploma and above	89	11.3

Paternal education	Unable to read and write	202	25.5
	Able to read and write	212	26.8
	Primary	271	34.3
	Secondary	44	5.6
	Diploma and above	62	7.8

Living with	Parents	253	32.0
	Relatives	224	28.3
	Peers	172	21.7
	Alone	142	18.0

Family structure	Joint	253	32.0
	Nuclear	538	68.0

Age of menarche	<121 years	195	24.7
	12–15 years	535	67.6
	>15 years	61	7.7

^*∗*^Others refer to Siltie, Wolayita, and Tigrie.

**Table 2:** Perception regarding menstruation among adolescent high school girls in Gedeo zone, Southern Ethiopia, 2019 (*N* = 791).

Question		Strongly disagree (0) (%)	Disagree (1) (%)	Agree (2) (%)	Strongly agree (3) (%)
*Menses*					
1	It is a normal phenomena	8.0	5.9	10.4	75.5
2	It is unique to females	24.7	17.7	6.8	50.8
3	It is a lifelong process	27.1	16.6	6.2	50.2
4	It will be stopped after initiation of sexual intercourse	31.6	13.5	15.3	18.6
5	It is a sign of conception	61.8	11.0	12.4	14.8
6	It has foul smell	13.5	13.1	13.8	59.5
7	It is pathological	58.9	8.05	11.8	21.4

*Sources*					
8	Uterus	39.1	52	23.1	32.6
9	Bladder	41.8	8.3	18.5	31.4
10	Vagina	22.8	18.5	11.5	47.3
11	Abdomen	40.3	13.5	20.5	25.7

*Causes*					
12	Hormonal	33.9	10.7	18.6	36.8
13	Diseases	51.8	11.6	16.3	20.7
14	Curse	51.1	10.5	8.7	27.7

*What is good to be done during menstruation*					
15	Not allowed touch others during menstruation period	51.1	11.9	22.5	14.5
16	Not allowed to go to kitchens during menses	53.2	12.8	13.7	20.4
17	It is not good to discuss about menses	50.1	10.7	13.3	25.9
18	Activities done by menstruating woman are not blessed	39.6	15.7	22.4	22.4
19	Being free from menses is a fate	37.7	17	22.3	22.5

**Table 3:** Bivariable and multivariable analysis of factors associated with perception regarding menstruation among adolescent high school girls in Gedeo zone, Southern Ethiopia (*N* = 791).

Variables	Categories	Level of perception		COR 95% CI	AOR 95% CI
		Good	Poor		
Residency	Town	165	383	1.00	1.00
	Rural	86	157	1.27 (0.92, 1.75)	1.33 (0.95, 1.87)

Birth order	First	86	199	0.60 (0.43, 0.85)	1.22 (0.79, 1.87)
	In between	105	221	2.53 (1.33, 4.83)	1.07 (0.71, 1.62)
	Last	60	120	1	1

Living with	Parents	83	170	1	**1**
	Relatives	80	144	0.87 (0.60, 1.28)	0.98 (0.66, 1.46)
	Peers	55	117	1.03 (0.68, 1.57)	1.07 (0.69, 1.64)
	Alone	33	109	1.61 (1.61, 2.57)	1.75 (1.07, 2.85)^*∗*^

Maternal education	Unable to read and write	64	214	3.91 (2.37, 6.46)	4.03 (2.41, 6.74)^*∗∗∗*^
	Able to read and write	42	96	2.6 (1.54, 4.64)	2.60 (1.48, 4.57)
	Primary	67	146	2.55 (1.53, 4.23)	2.47 (1.47, 4.14)
	Secondary	30	43	1.67 (0.98, 3.13)	1.74 (0.91, 3.29)
	Diploma and above	48	41	1.00	1

Age at menarche	<12 years	169	366	1.62 (0.76, 3.41)	2.07 (1.02, 4.24)^*∗∗*^
	12–15 years	69	126	1.82 (0.43, 3.96)	1.19 (0.82, 1.73)
	>15 years	13	48	1	1

^*∗*^ *p* value <0.05, ^*∗∗*^*p* < 0.01, and ^*∗∗∗*^*p* < 0.001.

## Data Availability

All the data included in the manuscript have been included in the form of tables. The deidentified raw data can be accessed up on request from the author ZB through zelalembe45@gmail.com.
